# Characterization and longitudinal assessment of daily quality assurance for an MR‐guided radiotherapy (MRgRT) linac

**DOI:** 10.1002/acm2.12735

**Published:** 2019-10-21

**Authors:** Kathryn E. Mittauer, David A.P. Dunkerley, Poonam Yadav, John E. Bayouth

**Affiliations:** ^1^ Department of Human Oncology School of Medicine and Public Health University of Wisconsin‐Madison Madison WI USA; ^2^ Department of Radiation Oncology Miami Cancer Institute Baptist Health South Florida Miami FL USA

**Keywords:** daily QA, MR guidance, MR linac QA, MR/RT isocenter, MRgRT, MR‐guided radiotherapy

## Abstract

**Purpose:**

To describe and characterize daily machine quality assurance (QA) for an MR‐guided radiotherapy (MRgRT) linac system, in addition to reporting a longitudinal assessment of the dosimetric and mechanical stability over a 7‐month period of clinical operation.

**Methods:**

Quality assurance procedures were developed to evaluate MR imaging/radiation isocenter, imaging and patient handling system, and linear accelerator stability. A longitudinal assessment was characterized for safety interlocks, laser and imaging isocenter coincidence, imaging and radiation (RT) isocentricity, radiation dose rate and output, couch motion, and MLC positioning. A cylindrical water phantom and an MR‐compatible A1SL detector were utilized. MR and RT isocentricity and MLC positional accuracy was quantified through dose measured with a 0.40 cm^2^ x 0.83 cm^2^ field at each cardinal angle. The relationship between detector response to MR/RT isocentricity and MLC positioning was established through introducing known errors in phantom position.

**Results:**

Correlation was found between detector response and introduced positional error (N = 27) with coefficients of determination of 0.9996 (IEC‐X), 0.9967 (IEC‐Y), 0.9968 (IEC‐Z) in each respective shift direction. The relationship between dose (Dose_MR/RT+MLC_) and the vector magnitude of MLC and MR/RT positional error (Error_mag_) was calculated to be a nonlinear response and resembled a quadratic function: Dose_MR/RT+MLC_[%] = −0.0253 Error_mag_ [mm]^2^ − 0.0195 Error_mag_ [mm]. For the temporal assessment (N = 7 months), safety interlocks were functional. Laser coincidence to MR was within ±2.0 mm (99.6%) and ±1.0 mm (86.8%) over the 7‐month assessment. IGRT position–reposition shifts were within ±2.0 mm (99.4%) and ±1.0 mm (92.4%). Output was within ±3% (99.4%). Mean MLC and MR/RT isocenter accuracy was 1.6 mm, averaged across cardinal angles for the 7‐month period.

**Conclusions:**

The linac and IGRT accuracy of an MR‐guided radiotherapy system has been validated and monitored over seven months for daily QA. Longitudinal assessment demonstrated a drift in dose rate, but temporal assessment of output, MLC position, and isocentricity has been stable.

## 1 INTRODUCTION

Image‐guided radiation therapy (IGRT) has enabled high accuracy and precision of treatment delivery through the use of imaging performed before and/or during treatment. Historically, on‐board imaging capabilities have been limited to kV and/or MV imaging modalities. Recently, magnetic resonance imaging (MRI) has been incorporated into radiotherapy treatment units with MR‐guided radiotherapy (MRgRT) systems.

The emergence of MRgRT systems poses a new set of challenges when implementing existing quality assurance (QA) equipment and procedures that have been utilized with x‐ray‐based IGRT systems. Daily QA is performed on conventional linac accelerators utilizing either an on‐board imaging array or a detector array for convenient, robust dosimetric verification without the need of handling individual ionization chamber(s).^1^ A current commercial MRgRT linac (MRIdian™, ViewRay Inc., Cleveland, OH, USA) does not include an on‐board, x‐ray‐based detector for dosimetric verification. Additionally, no MR‐compatible detector arrays have been developed that are visible in MR imaging.

Another unique challenge of MRgRT is the ability to verify IGRT isocenter coincidence to radiation isocenter. Since conventional linear accelerators have a single on‐board detector that is compatible with both imaging and radiation source, simple localization of a phantom through the two modalities (i.e., MV/kV) is used to verify coincidence, while MRgRT systems contain no such detector. Currently, no method of verification of isocentricity has been reported in the literature. Current solutions to evaluate MR/RT isocentricity for MRgRT systems have been practically carried out with film enclosed by a water phantom using a star shot irradiation technique, eliminating real‐time information and impractical for a daily QA technique.^2^

Daily QA guidance has been previously established for conventional linear accelerators and MR imaging systems.^3^, ^4^, ^5^, ^6^, ^7^, ^8^ The daily QA tests recommended in TG142, TG40, and MPPG 8a are designed to maintain safety, accurate patient localization, and dosimetric output by monitoring parameters which can impact treatment goals.^3^, ^5^, ^6^ However, there is no existing literature or guidance for routine QA on the two integrated systems to ensure consistent and safe operation and accurate treatment delivery using MRgRT.

In this study, we have developed and implemented an efficient and sensitive QA procedure to characterize the MR imaging/radiation isocenter alignment, spatial fidelity of imaging and patient handling systems, and the performance of the linear accelerator on an MRgRT system for routine daily QA. A method for real‐time characterization of the MR/RT isocentricity is established through exploiting the sensitivity of the penumbra position across a large detector relative to the field size. As such, our method utilizes an MR‐compatible A1SL detector (Standard Imaging Inc., Middleton, WI, USA) with an active volume of 4.4 mm length and 4 mm diameter placed within a cylindrical water phantom with a 0.40 cm^2^ x 0.83 cm^2^ field size to optimize the spatial sensitivity of MR/RT isocentricity and MLC position. This work is the first reporting of a daily QA procedure for an MR‐guided radiotherapy system. The sensitivity of our methods has been characterized through introducing known errors and/or through comparing to established QA procedures. Lastly, we describe the first reporting of the longitudinal assessment of the dosimetric and mechanical accuracy of a commercial MRgRT linac over a 7‐month period of clinical operation.

## 2 MATERIALS AND METHODS

### 2.1 ViewRay MRIdian linac

The MRIdian linac, previously described by Hill and Mittauer, consists of a gantry‐mounted 6 MV linear accelerator and a 0.345 T MRI scanner.^2^ The linac produces a 6‐MV flattening filter free beam with a nominal dose rate of 600 MU/min. Beam collimation is achieved using the RayZR™ MLCs, consisting of a set of two banks of MLCs, stacked and double focused, and offset by one‐half leaf width, eliminating the need for MLC tongue and groove design.^9^ With a 90 cm SAD, the MLCs project to a maximum field size of 27.4 cm^2^ x 24.07 cm^2^ at isocenter with individual leaf width projections of 8.3 mm.

### 2.2 Overview of phantom and QA procedures

**Figure 1:**
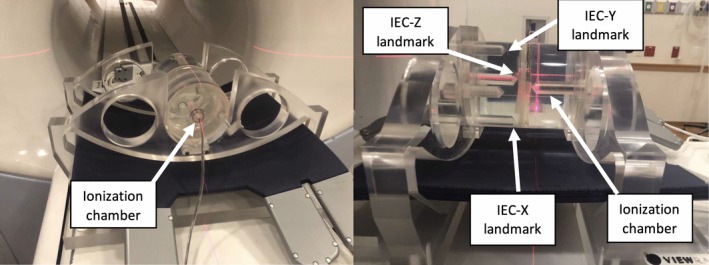
Cylindrical water phantom with MR‐compatible A1SL ionization chamber (left). Additional periphery ionization chamber locations used as landmarks for IGRT position–reposition evaluation (right). Anterior‐positioned torso coil not pictured.

An MR‐compatible A1SL detector (active volume of 4.4 mm length and 4 mm diameter) within a cylindrical water phantom (ViewRay Inc., Cleveland, OH, USA) shown in Fig. 1 was utilized for this study. The phantom is filled with distilled water to enable MR imaging capabilities. The phantom includes scribed locations for laser alignment which are coincident with the centroid of the active volume of the ionization chamber. There are four additional chamber positions located at the periphery of the cylindrical phantom. The phantom is indexed to the table through two mounting brackets, with a cutout for the posterior‐oriented torso receiver coils.

An overview of the daily QA procedures is listed in Table 1, categorized by dosimetry, mechanical and imaging, and safety tests, along with the applicable tolerance from TG142 based on SBRT/SRS specifications. A description of the QA method and the technique used to characterize the method is also listed in Table 1.

**Table 1:** Overview of daily QA procedures, tolerances, technique, and characterization method.

Procedure	Tolerance	Description of technique	Method characterization
Dosimetry			
Output constancy	3%	10.04 cm^2^ x 9.96 cm^2^ field output	TLD measurements and TG51
Dose rate constancy	2%	10.04 cm^2^ x 9.96 cm^2^ field MU/ time from UI	–
Mechanical & imaging			
Laser and imaging isocentricity	1 mm	Daily QA phantom setup to scribe lines	Registration of phantom image
Radiation and imaging isocentricity	1 mm	0.40 cm^2^ x 0.83 cm^2^ field dose at cardinal angles	Characterization of phantom shift vs. dose
Patient position–reposition accuracy	1 mm	Apply known shifts to phantom	Imaging of phantom landmarks
MLC positional accuracy	1 mm	0.40 cm^2^ x 0.83 cm^2^ field dose at cardinal angles	Characterization of phantom shift vs. dose
Imaging coil functionality	Functional	–	–
Safety			
Door interlocks	Functional	–	–
Radiation area monitor	Functional	–	–
Beam‐off/Radiation interrupt	Functional	–	–
In‐room camera	Functional	–	–
In‐room audio	Functional	–	–
Headphone audio	Functional	–	–
Panic bulb	Functional	–	–

### 2.3 Safety functionality

Implemented safety checks include functionality of patient monitoring, radiation monitoring, beam interruption, and door interlocks. Patient monitoring is verified for audio/visual communication devices, including the panic bulb and audio headphones. Radiation monitoring equipment, radiation interrupts, and interlocks are tested during beam delivery. The radiation area monitor and the control panel beam on indicator are visually confirmed to be operational. Functionality of the treatment vault door to prevent radiation generation with the door open and to interrupt radiation delivery of the beam when opened is independently verified. Additional door interlocks on the radiofrequency (RF) shielded doors designed to reduce RF interference during imaging are verified to be operational.

### 2.4 MR spatial fidelity and phantom localization

The room lasers define a virtual isocenter located −155 cm in IEC‐Y direction from the MRIdian MR/RT isocenter. To verify that the lasers are coincident to this virtual isocenter, the phantom is initially aligned to the in‐room lasers using the external scribe marks, translated +155 cm in the IEC‐Y direction, and then localized based on MR imaging. A balanced steady‐state–free precession sequence (TrueFISP) MR scan is acquired in 65 s with a 1.5 mm^3^ x 1.5 mm^3^ x 1.5 mm^3^ resolution over 45 cm^3^ x 23 cm^3^ x 26 cm^3^ field of view for the daily setup MR scan and the simulation reference MR scan, that is, the primary dataset used for the treatment plan generation. Maximum spatial distortion of the MRIdian TrueFISP sequence is <1 mm within 5 cm of isocenter.^10^ Localization is then achieved through manual alignment of the chamber holder about the active volume of interest for the A1SL ionization chamber. Phantom shifts are recorded as the difference between laser and imaging isocenters for the IEC‐X, IEC‐Y, and IEC‐Z dimensions.

**Figure 2:**
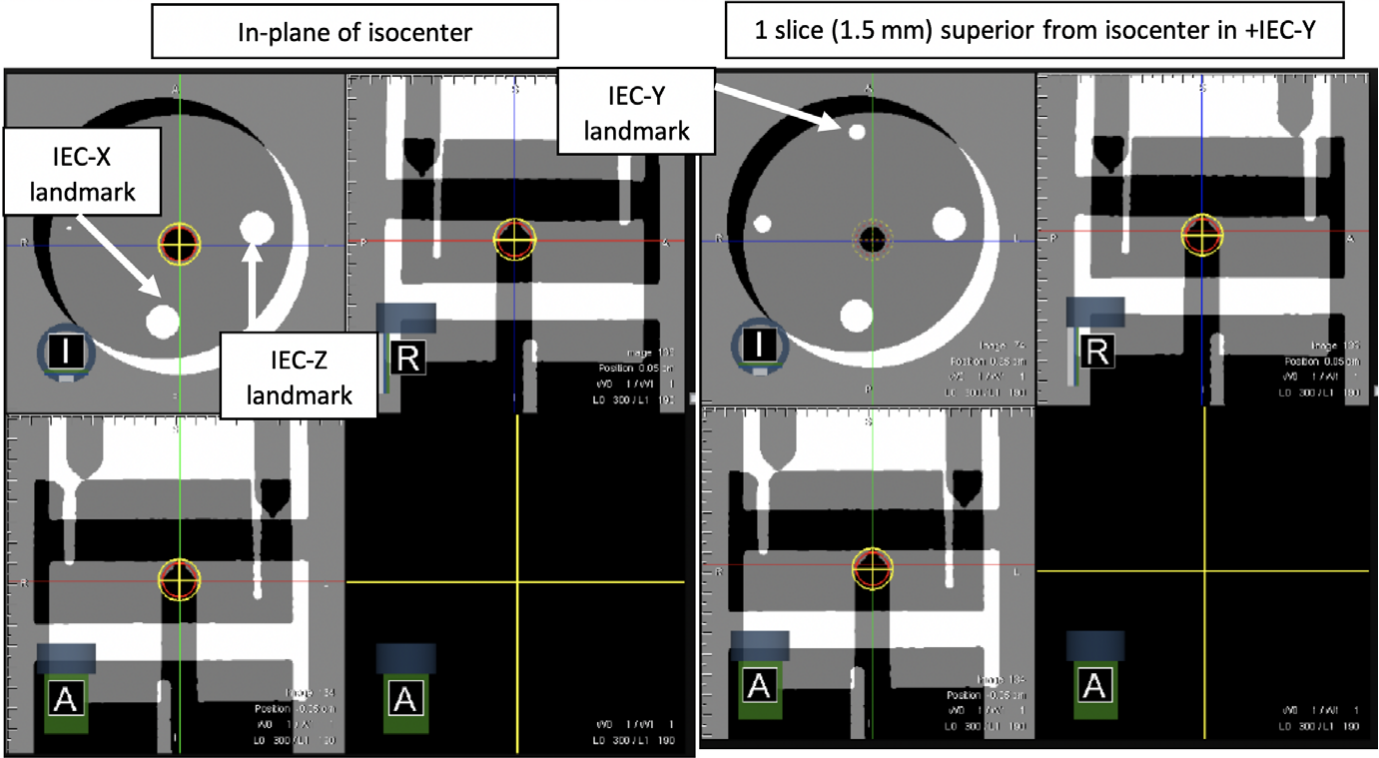
IGRT position–reposition alignment showing the phantom position postcouch offsets applied. Verification of couch and IGRT positioning accuracy performed with visualization of landmarks using greyscale (image view) and inverse greyscale image (positioning scan) values with window = 1.

Postonline couch shifts for initial phantom localization, an IGRT position–reposition test is performed in accordance with TG142. Here, a known shift is introduced of −0.75 cm (IEC‐X), −4.9 cm (IEC‐Y), and +0.75 cm (IEC‐Z), and the phantom is subsequently reimaged. Couch movement and geometric spatial fidelity of the MR imaging is assessed using the known physical landmarks within the phantom (Fig. 2). The image of the shifted phantom ideally places the edges of the peripheral chamber inserts in a known geometry. Specifically, at the image volume origin, the IEC‐X landmark intersects the edge of the sagittal plane in the axial view, and the IEC‐Z landmark intersects the edge of the coronal plane in the same axial view (Fig. 2). The IEC‐Y landmark is visually verified by identifying the beginning of its edge on the superior‐adjacent axial slice (i.e., located 1.5 mm from isocenter in +IEC‐Y). Postvisual verification of the positioning offset by known landmarks, the phantom is shifted back to isocenter and reimaged. Sensitivity of the visual inspection of the known offset is limited by the voxel resolution (1.5 mm isotropic). The phantom is localized again, and any residual registration of alignment for both the offset position and aligned position is taken as the error of the position–reposition localization.

### 2.5 Dosimetry, MLC, and MR/RT isocentricity

**Figure 3:**
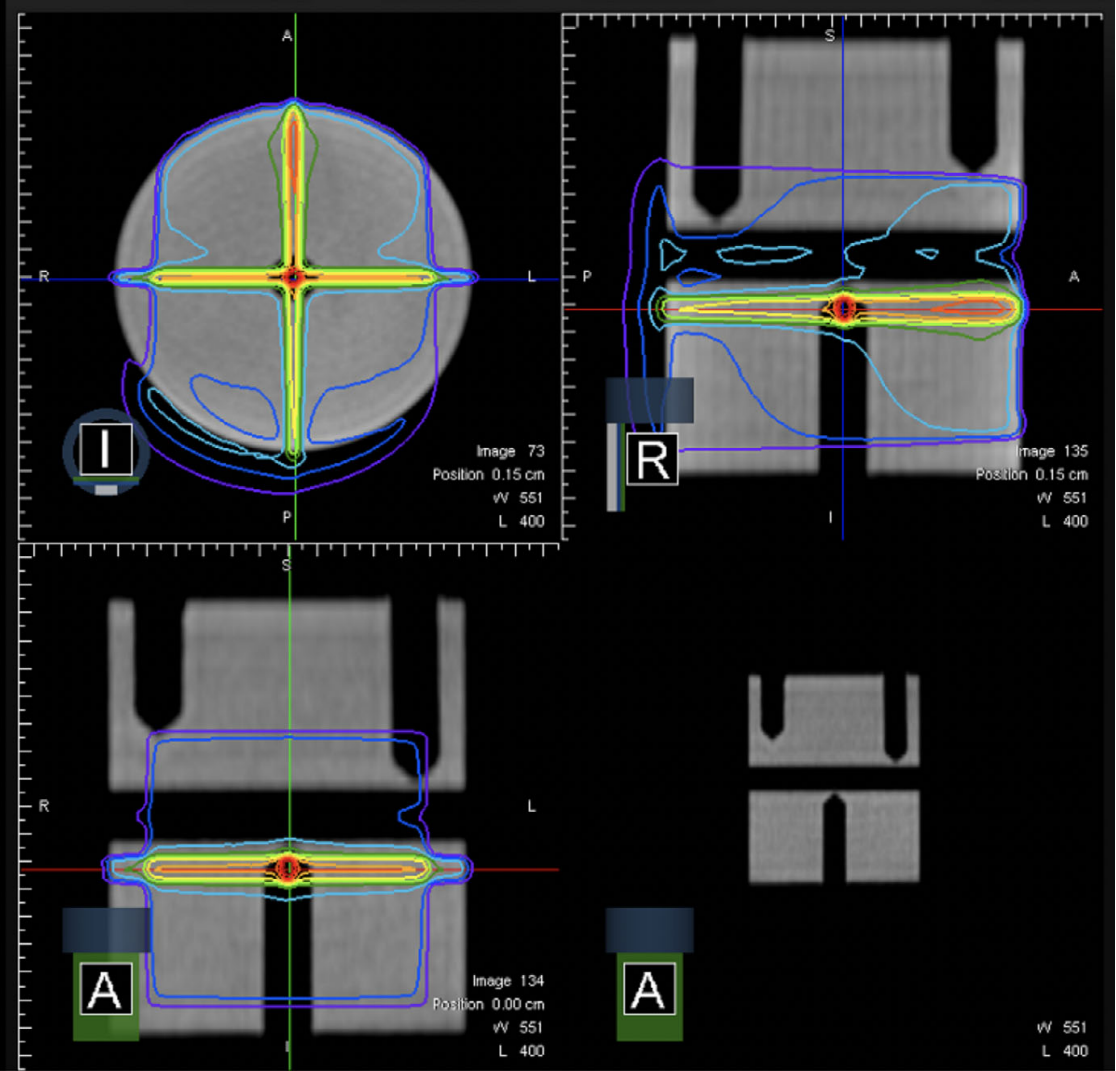
Dose distribution for five‐field 3D conformal daily QA plan with 10.04 cm^2^ x 9.96 cm^2^ to measure the dosimetric output and a 0.40 cm^2^ x 0.83 cm^2^ to characterize spatial accuracy of MR/RT isocentricity and MLC at each cardinal gantry angle. Active volume of ionization chamber denoted as region of interest.

Postphantom localization of the chamber active volume with isocenter, a five‐field 3D conformal treatment plan (Fig. 3) is delivered. The plan includes a 10.04 cm^2^ x 9.96 cm^2^ field delivered with the gantry at zero degrees (G0, IEC 1217) to measure the dosimetric output and a 0.40 cm^2^ x 0.83 cm^2^ field at each cardinal angle characterizes the spatial accuracy of MR/RT isocentricity and MLC positional accuracy at each cardinal gantry angle. This field size was selected to optimize spatial sensitivity with the A1SL active volume (described above). Ionization values are converted to dose based on chamber and electrometer coefficient and temperature and pressure values. Dose is monitored as the ratio with respect to baseline dose for each field to eliminate reduction of sensitivity from averaging across multiple gantry angles. To monitor the linac constancy, dose rate is calculated as the delivered MU over the recorded delivery time from the UI of the treatment planning and delivery system (TPDS) for the 10.04 cm^2^ x 9.96 cm^2^ field.

Reference dose was calculated using MRIdian's Monte Carlo dose calculation algorithm with magnetic field corrections, dose grid resolution of 2 mm^3^, and Monte Carlo uncertainty of 0.2%. Reference dose was taken for each of the five 3D conformal fields for respective baseline values. Reference dose was reported as a point of interest (POI) for 0.40 cm^2^ x 0.83 cm^2^ and a region of interest (ROI) approximating the active volume of the A1SL chamber for 10.04 cm^2^ x 9.96 cm^2^. The POI was selected for small fields, due to the limitation of TPS ROI delineation as whole voxel delineation. The reference dose to POI was calculated several times to estimate uncertainty due to random particle histories to the POI, dose discrepancies were <0.28% between repeated calculations. Retrospectively, the dose grid resolution was set to 1 mm^3^ to evaluate impact of 2 mm^3^ on the reference dose for the 0.40 cm^2^ x 0.83 cm^2^ fields, dose discrepancies were <0.20% between the dose grid resolutions.

### 2.6 Characterization of methods

The sensitivity of our methods has been characterized through introducing known errors and/or through comparing to established QA procedures. The relationship between detector response to MR/RT isocentricity and MLC positioning was established through introducing known errors in phantom position to simulate offsets in MR/RT isocentricity and MLC position.

The MLC positional accuracy and the MRI to RT isocentricity had been characterized on the institution's MRIdian, independent of the methods utilized for this study. Specifically, a five‐field star shot every 72° of gantry rotation with radiochromic film inserted into the cylindrical phantom (IEC‐X and IEC‐Z displacement) and film wrapped around the exterior of the cylindrical phantom (IEC‐Y displacement) demonstrated radiation coincidence to within 0.6 mm (radius) with phantom alignment based on MR imaging at G0. Additionally, the MRI isocenter walkout as a function of gantry rotation was characterized through 3D imaging of the cylindrical water phantom every 15° of gantry rotation and registering to the baseline image acquired at G0. The maximum magnitude of vector displacement of isocenter was found to be 1.4 mm, and at the cardinal angles found to be have vector displacement of 1.1 mm (G90), 1.4 mm (G180), 1.0 mm (G270) with respect to the phantom localization at G0. MLC positional accuracy at central axis and off axis was previously reported at our institution by Mittauer *et al*. using an ionization profiler and found to be within 0.06 ± 0.16 mm at baseline with no drift 0.00 ± 0.12 mm (n = 6 months) quantified using half beam block at cardinal angles about the central axis.^9^

To characterize this study's methods of detector response to MR/RT isocentricity and MLC positioning, a baseline measurement was first established at each cardinal angle with the active volume of ionization chamber centered with respect to isocenter, based on MRI. To simulate offset, the phantom was shifted in 1‐mm increments along the IEC‐X, IEC‐Y, and IEC‐Z axes (IEC 1217 convention) independently and dose delivered with the 0.40 cm^2^ x 0.83 cm^2^ field was recorded for each shifted position.^11^ Simultaneous shifts in two or more directions were also reported. The dose measured from introduced shift was normalized to the dose measured with the chamber positioned at isocenter. The relationship between dose and vector magnitude of MLC and MR/RT positional error was quantified.

The dosimetric output using the daily QA procedure was benchmarked by comparison to measurements performed with monthly QA using TG51 protocol in a water tank, in addition to an independent output verification through an Accredited Dosimetry Calibration Laboratory (ADCL) service with TLD irradiation.^12^ Here, six TLDs were irradiated on MRIdian under reference conditions with a dose of 200 cGy at a depth of 10 cm, 90 cm SAD. The calibration TLDs were irradiated on an independent system, TrueBeam STx (Varian Medical Solutions Inc., Palo Alto, CA, USA), with a known dose delivered to the TLDs ranging from 180 to 220 cGy using the 6‐MV FFF beam to minimize spectral differences between the beams.

### 2.7 Longitudinal assessment

**Figure 4:**
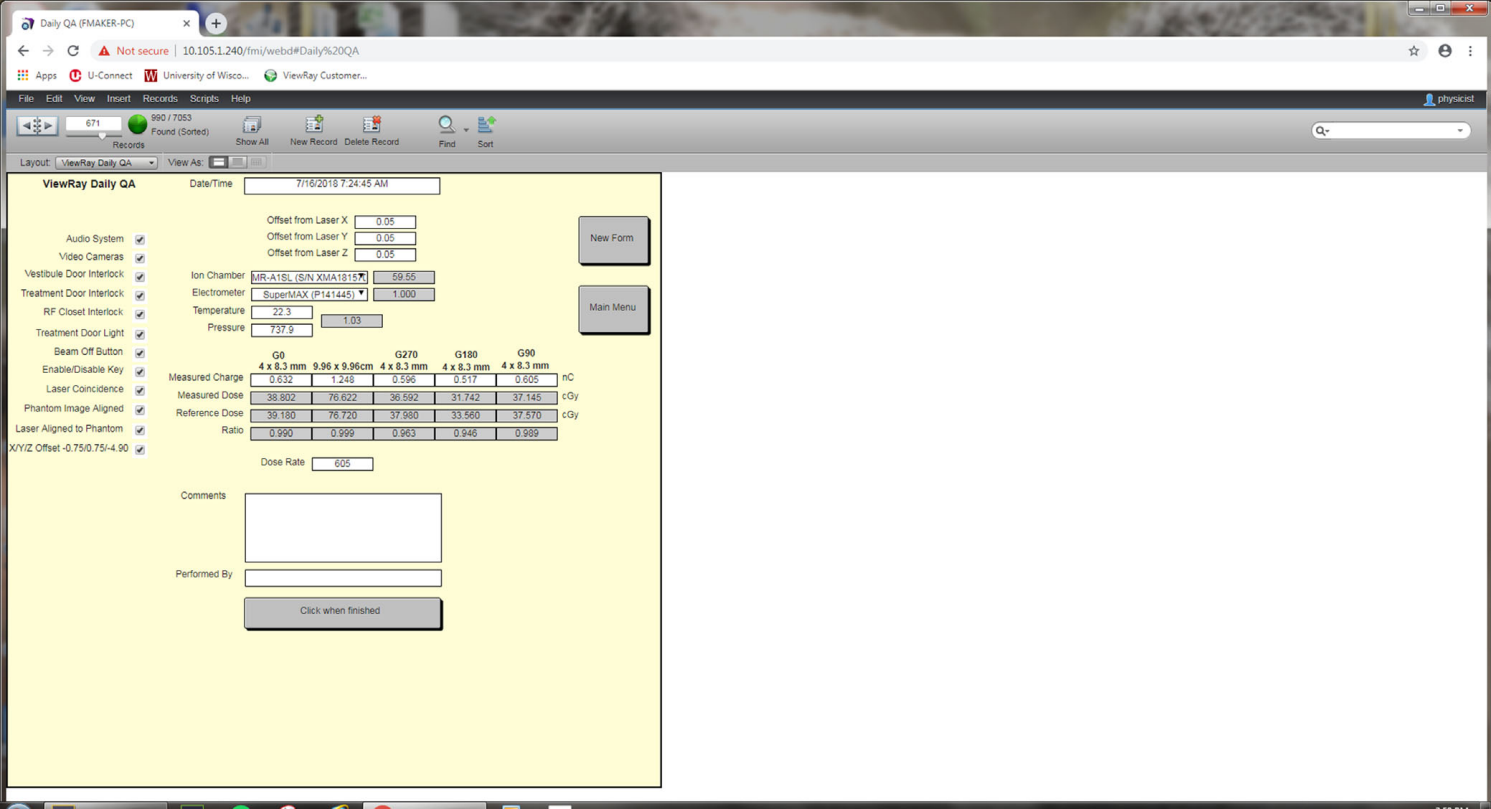
Institutional data record for MRIdian daily QA electronic database, utilizing the FileMaker Pro application.

A longitudinal assessment of the daily QA performance on MRIdian linac was evaluated over a 7‐month period (June 4, 2018 to January 12, 2019, N = 166 measurements). FileMaker Pro, a cross‐platform database manager which allows for the creation of graphical user interfaces and forms for data input, was implemented to record the daily QA results (Fig. 4). Functional tests were evaluated as a binary pass–fail test. Quantitative data were recorded for laser and imaging isocentricity, IGRT position–reposition, MR/RT isocentricity and MLC accuracy as measured by dose of 0.40 cm^2^ x 0.83 cm^2^ at each cardinal angle, dose and dose rate for the 10.04 cm^2^ x 9.96 cm^2^. A one‐way ANOVA was used to determine if a statistical difference existed across the relative output of the 0.40 cm^2^ x 0.83 cm^2^ field size across the four cardinal angles in the longitudinal assessment.

## 3 RESULTS

**Figure 5:**
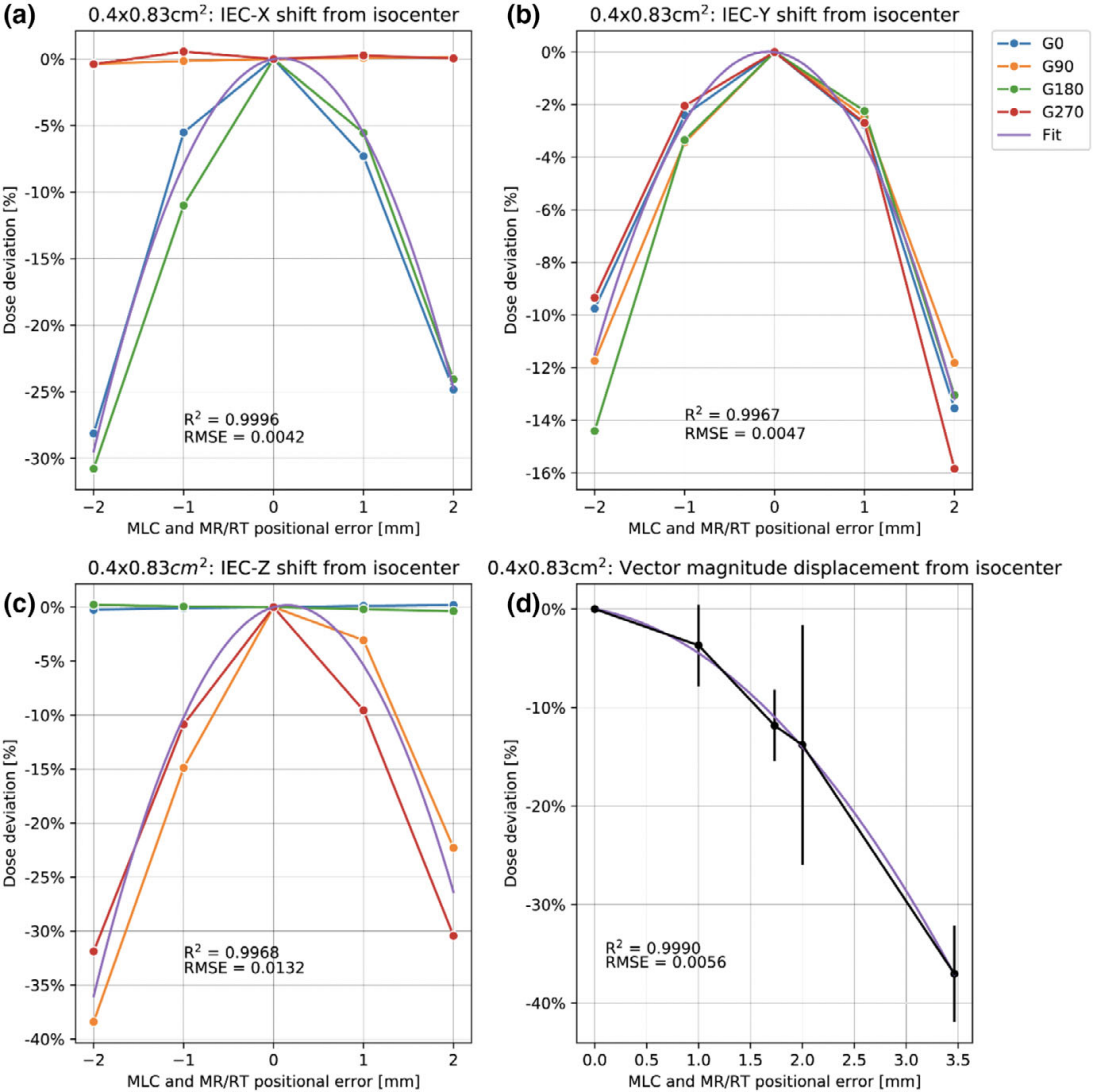
The relationship between dose (Dose_MR/RT+MLC_) and the MLC and MR/RT positional error in IEC‐X (Error_IEC‐X_, a), IEC‐Y (Error_IEC‐Y_, B), and IEC‐Z (Error_IEC‐Z_, C) directions in addition to the vector magnitude of MLC and MR/RT positional error (Error_mag_, D) across all three directions.

The sensitivity of the 0.40 cm^2^ x 0.83 cm^2^ field (N = 27 introduced errors) is displayed in Fig. 5. Percentage difference in dose from isocenter (Dose_MR/RT+MLC_) with respect to vector magnitude of phantom positional error (i.e., surrogate of MLC and MR/RT positional error) is quantified for a unidirectional positional error [Fig. 5(a)–5(c)] for each cardinal angle and a positional error in two or more directions [Fig. 5(d)] averaged across all gantry angles with error bars representing ±1 SD.

The relationship between dose (Dose_MR/RT+MLC_) and the MLC and MR/RT positional error in IEC‐X [Error_IEC‐X_, eq. (1)], IEC‐Y [Error_IEC‐Y_, eq. (2)], and IEC‐Z [Error_IEC‐Z_, eq. (3)] directions in addition to the vector magnitude of MLC and MR/RT positional error [Error_mag_, eq. (4)] across all three directions was calculated to be a nonlinear response and resembled a quadratic function:

(1) $$\begin{array}{cc} {\text{Dose}}_{\text{MR}/\text{RT}+\text{MLC}}\left[\%\right]=-0.0677\;{\text{Error}}_{\text{IEC}-X}{\left[\text{mm}\right]}^{2} & \\ +0.0119\;{\text{Error}}_{\text{IEC}-X}\left[\text{mm}\right] \end{array}$$

(2) $$\begin{array}{cc} {\text{Dose}}_{\text{MR}/\text{RT}+\text{MLC}}\left[\%\right]=-0.0308\;{\text{Error}}_{\text{IEC}-Y}{\left[\text{mm}\right]}^{2} & \\ -0.0042\;{\text{Error}}_{\text{IEC}-Y}\left[\text{mm}\right] \end{array}$$

(3) $$\begin{array}{cc} {\text{Dose}}_{\text{MR}/\text{RT}+\text{MLC}}\left[\%\right]=-0.0780\;{\text{Error}}_{\text{IEC}-Z}{\left[\text{mm}\right]}^{2} & \\ +0.0241\;{\text{Error}}_{\text{IEC}-Z}\left[\text{mm}\right] \end{array}$$

(4) $$\begin{array}{cc} {\text{Dose}}_{\text{MR}/\text{RT}+\text{MLC}}\left[\%\right]=-0.0253\;{\text{Error}}_{\text{mag}}{\left[\text{mm}\right]}^{2} & \\ -0.0195\;{\text{Error}}_{\text{mag}}\left[\text{mm}\right] \end{array}$$

The coefficient of determination (R^2^) and root mean square error (RMSE) are displayed in Fig. 5 for the respective equations. Note that the measurements for shifts in the gun‐target direction were omitted from the quadratic fit for unidirectional positional errors only, that is, IEC‐X and IEC‐Z [Fig. 5(a) and 5(c)].

**Figure 6:**
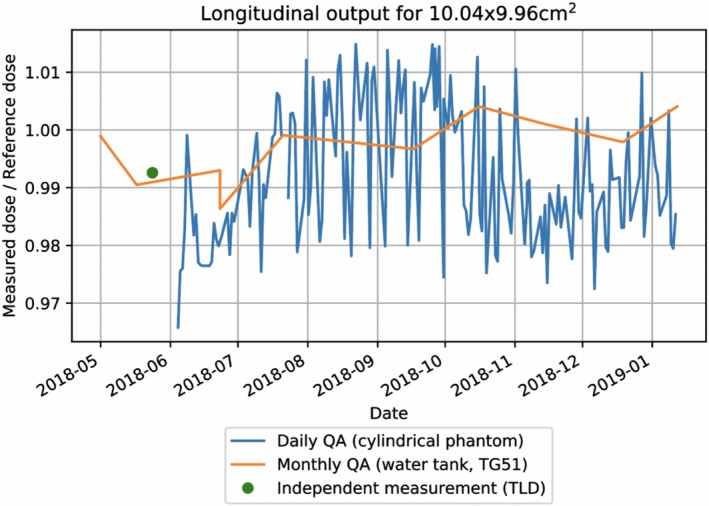
Comparison of dosimetric output stability for monthly QA (TG51 protocol in water tank), daily QA (cylindrical water phantom), and an independent output measurement (ADCL‐reported TLD irradiation).

Dosimetric output of the MRIdian linac for monthly QA (TG51 protocol in water tank), daily QA (cylindrical water phantom), and an independent output measurement (ADCL‐reported TLD irradiation) are displayed in Fig. 6 as a ratio of the measured dose to the expected dose from calibration, 1 cGy/MU. The mean dose to the TLDs reported by the ADCL at 0.992 ± 0.008 agreed with the institutional measurements of TG51 monthly QA measurements at 0.991, and the daily QA measurement at 0.992 measured on same date. The TLD measurement and the corresponding TG51 measurement on the same day were within 0.95% of the expected 1 cGy/MU, and the corresponding daily QA measurement was within 0.80%. The mean difference between all TG51 and daily QA measurements on days when both measurements were performed (N = 7) was 0.008 ± 0.01.

Figure 6 also displays the longitudinal performance of the MRIdian output over the seven months. The mean deviation in linac output was 0.992 ± 0.012 for daily QA and 0.997 ± 0.005 for monthly QA as measured by the TG51 protocol

**Figure 7:**
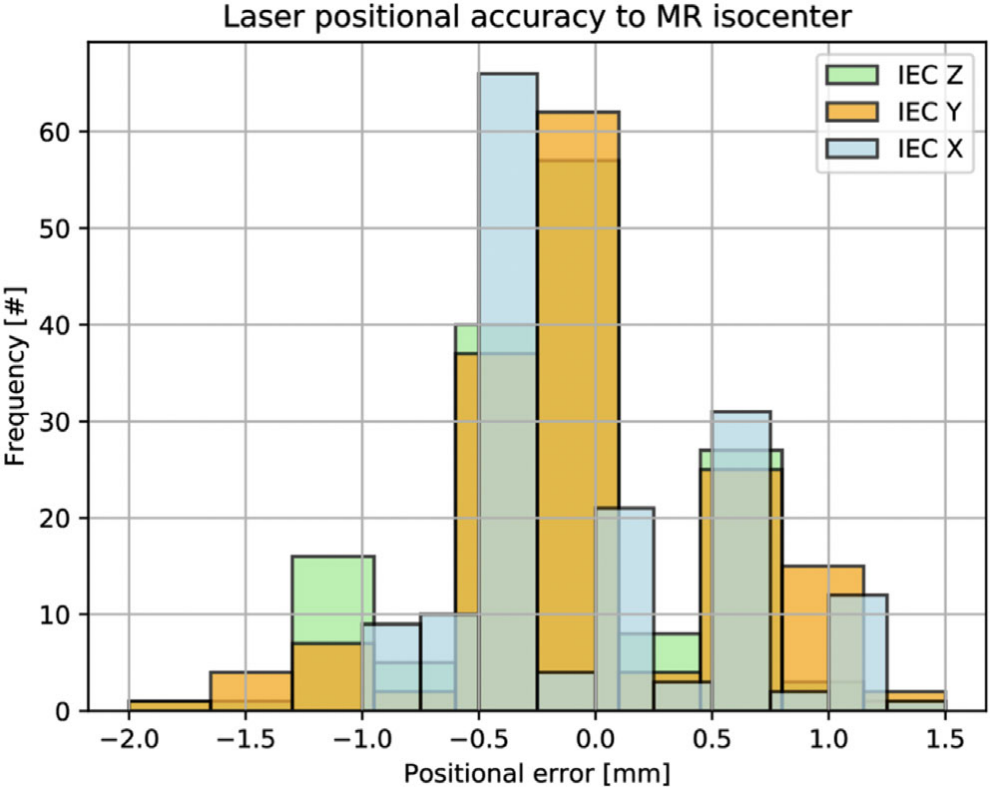
Histogram of longitudinal assessment of laser positional accuracy to MR isocenter coincidence.

The positional accuracy of the laser to MR imaging isocenter coincidence is displayed as a histogram in Fig. 7 over the 7‐month longitudinal assessment. Laser coincidence to MR was within ±2.0 mm for 99.6% of all measurements and within ±1.0 mm for 86.8% of all measurements. The mean laser positional error to MR imaging was −0.09 ± 0.56 mm (IEC‐X), −0.03 ± 0.58 mm (IEC‐Y), and −0.15 ± 0.53 mm (IEC‐Z).

**Figure 8:**
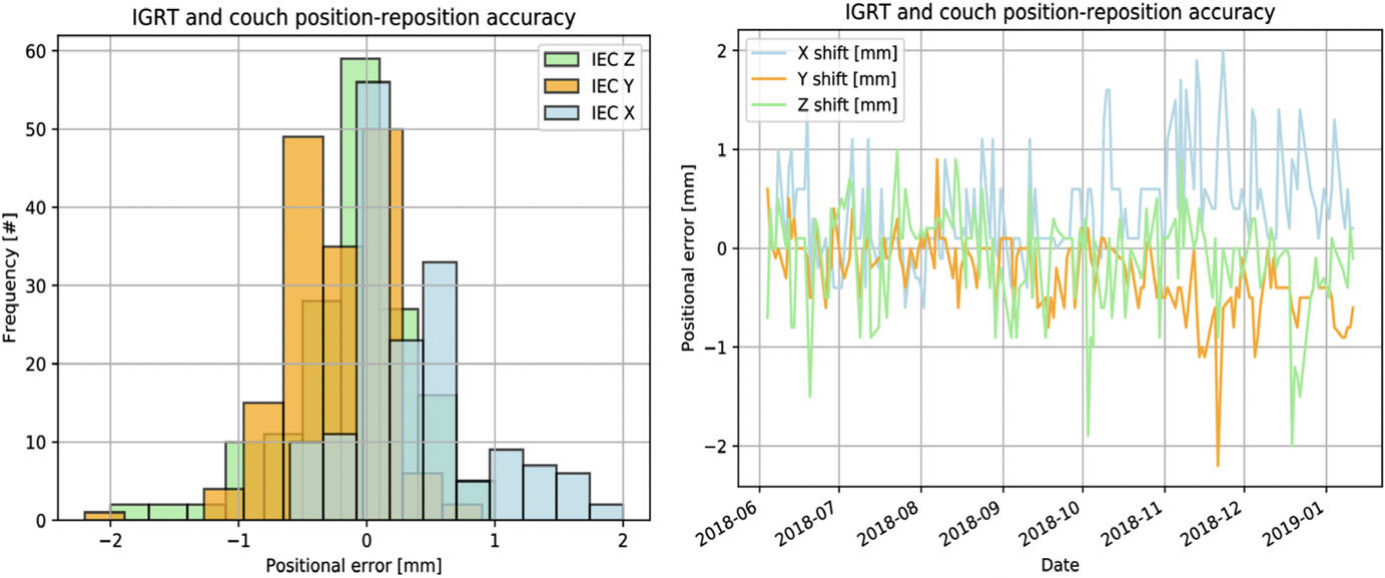
Histogram (left) and line plot (right) of IGRT and couch position–reposition accuracy.

The IGRT and couch position–reposition accuracy is shown in Fig. 8. The position–reposition accuracy was 0.38 ± 0.52 mm (IEC‐X), −0.25 ± 0.38 mm (IEC‐Y), and −0.11 ± 0.51 mm (IEC‐Z). IGRT position–reposition accuracy was within ±2.0 mm for 99.4% and ±1.0 mm for 92.4% of all measurements on the MRIdian linac.

**Figure 9:**
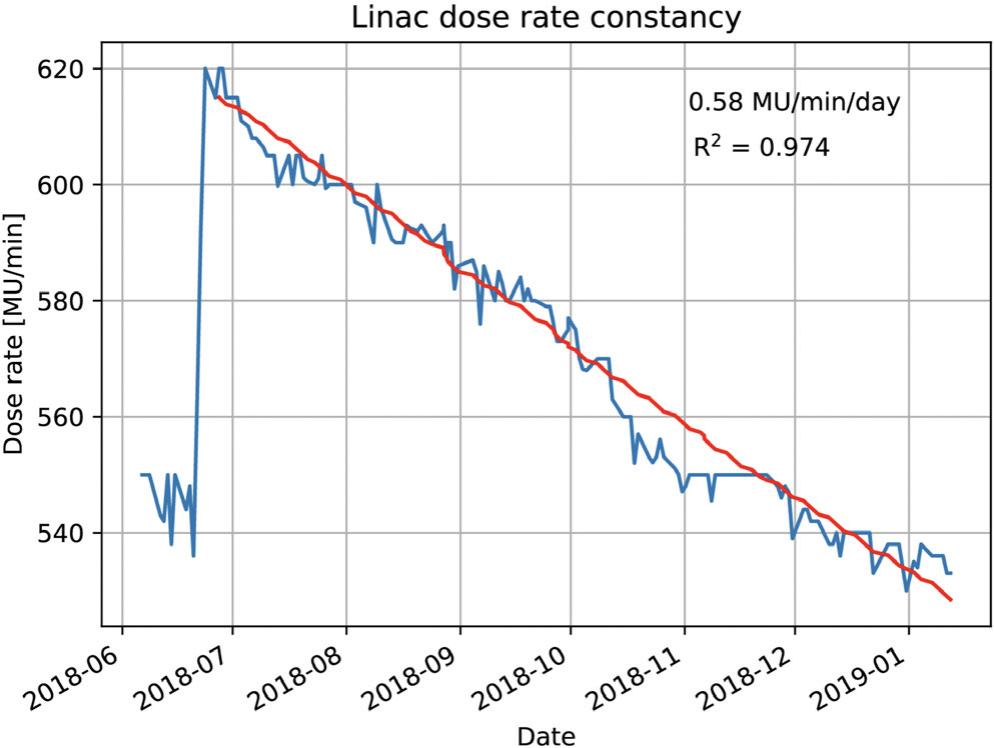
Longitudinal dose rate performance of the MRIdian linac with PRF adjustment performed on June 23, 2018.

The longitudinal assessment of dose rate of the MRIdian linac is displayed in Fig. 9. A steady decrease, from 630 MU/min at initial installation to 545 MU/min in July 2018, prompted an adjustment in the pulse repetition frequency (PRF) from 161 Hz to 183 Hz. Post‐PRF adjustment a decrease in dose rate continued to be observed from the adjusted dose rate. A linear fit to this portion of the data indicates a decrease in dose rate of 0.58 MU/min/day (R^2^ = 0.974).

**Figure 10:**
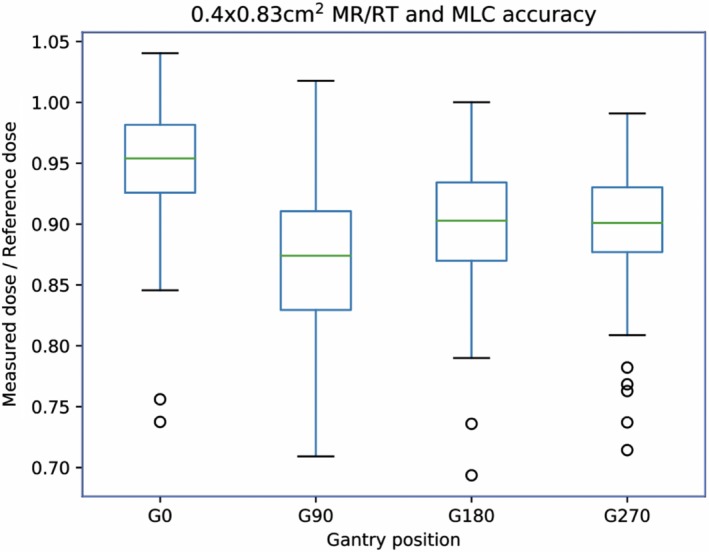
MR/RT isocentricity and MLC positional accuracy of the MRIdian linac for 0.40 cm^2^ x 0.83 cm^2^ field at cardinal gantry angles over longitudinal evaluation.

To evaluate the spatial accuracy of the MLC and MR/RT isocentricity of the MRIdian linac, the dosimetric change for a 0.40 cm^2^ x 0.83 cm^2^ field at each cardinal gantry angle is displayed in Fig. 10 as a box plot. The average and standard deviation of the ratio of measured to reference dose was 0.95 ± 0.05 (G0), 0.87 ± 0.06 (G90), 0.90 ± 0.05 (G180), and 0.90 ± 0.05 (G270). Solving the equation from the fit of vector magnitude positional shifts in Fig. 5(d), the mean MR/RT and MLC positional error of the MRIdian linac was 1.1 mm (G0), 1.9 mm (G90), 1.6 mm (G180), and 1.6 mm (G270) over the 7‐month longitudinal assessment. There is a statistical difference (p < 0.001) observed among the four cardinal angles, likely attributed to small changes in radiation isocenter or gravity‐induced MLC effects with gantry rotation.

All safety QA procedures found in Table 1 passed functionality on a daily basis over the longitudinal assessment.

## 4 DISCUSSION

The clinical efficacy of MR guidance has previously been shown by the MRgRT community.^13^ The superior soft tissue visualization combined with real‐time tracking capabilities has enabled greater confidence in the treatment delivery allowing for a reduction in the planning target margin compared to CT‐based IGRT modalities.^13^ However, the temporal assessment of a clinical MRgRT system or implementation of daily QA has yet to be reported. Current commercial daily QA equipment and guidance criteria are limited to CT‐based IGRT modalities may not be applicable to MRgRT systems due to equipment incompatibility in a magnetic field and/or fundamental differences in the clinical utility of the technology. In this study, we describe a novel daily QA procedure that exploits the spatial sensitivity of the penumbra to evaluate the MLC positional accuracy and MR/RT isocentricity for an efficient, robust daily QA procedure. In addition, we report the first evaluation of the longitudinal assessment of a clinical MRgRT linac system in terms of IGRT spatial fidelity and linac integrity.

Our technique has allowed TG142 criteria to be applicable and quantified on a daily basis. Currently, no guidance has been established for best practices and/or tolerances for routine QA of clinical MRgRT systems. For our evaluation, we applied TG142 tolerances for CT‐based IGRT systems where applicable. Daily QA techniques on CT‐based modalities often employ on‐board imaging systems.^1^, ^14^ However, the implementation of x‐ray‐based detectors may not be applicable to MRgRT with some commercial MRgRT systems not having an onboard x‐ray detector (i.e., MRIdian), therefore an external array or ionization chamber is necessary. Our technique has employed one phantom in combination with a single ionization chamber. However, care must be taken with daily handling of an ionization chamber and triaxial cable.

Implementation of the daily output was benchmarked by comparing two independent procedures: TG51 protocol in water tank, and TLD service reported by the ADCL. The output as measured with daily QA procedure was in line with both TG51 procedure, and the independent TLD readings within 0.15%. Overall dosimetric output of the MRIdian linac was very stable over the first 7 months of clinical use and within tolerance of TG142 criteria of 3% for 99.4% of daily measurements (Fig. 6). Small fluctuations may be attributed to the machine and/or chamber and electrometer not fully warmed up prior to output measurement.

One observed drift over the temporal assessment of the MRIdian linac was a systematic decrease in dose rate from time of initial installation (Fig. 9). The dose rate was intentionally increased in July 2018 through changing the PRF from 161 Hz to 183 Hz. The increase of PRF was noted to have a small increase in the linac dark current, which continues to be monitored on a monthly basis.

The MR/RT isocentricity and MLC positional accuracy as measured with 0.40 cm^2^ x 0.83 cm^2^ field at each cardinal angle was benchmarked for sensitivity through introducing known errors across IEC‐X, IEC‐Y, IEC‐Z for each cardinal angle (Fig. 5). As such, implementation of this small field allowed for characterization of not only shifts due to MR/RT offset but also MLC positional error as a function of gantry angle. The overall sensitivity was found to be 5.6% (1 mm) and 24.7% (2 mm) for IEC‐X [Fig. 5(a)], 3.5% (1 mm) and 13.2% (2 mm) for IEC‐Y [Fig. 5(b)], and 5.4% (1 mm) and 26.4% (2 mm) for IEC‐Z [Fig. 5(c)] for unidirectional errors and 4.5% (1 mm) and 14.0% (2 mm) for the vector magnitude of error [Fig. 5(d)]. Shifts along the gun‐target direction were relatively insensitive due to negligible change in photon fluence as a function of inverse square of small distance changed from the source to detector [Fig. 5(a), G90 and G270; Fig. 5(c), G0 and G180]. The IEC‐X and IEC‐Z directions [Fig. 5(a) and 5(c)] were found to be more sensitive in comparison to the IEX‐Y direction [Fig. 5(b)]. This was due to the resolution of field size is limited by MLC leaf width (i.e., 4.15 mm) across the IEC‐Y direction. The field size in IEC‐Y direction was selected as 8.3 mm to allow for symmetric evaluation about isocenter, as the MRIdian central leaves of 4.15 mm are split above and below isocenter with respect to IEC‐Y. Therefore, the IEC‐Y field length (8.3 mm) is larger than the active length (4.4 mm) leading to the observed reduction in sensitivity. For the multidirectional shifts [Fig. 5(d)], the overall reduction in sensitivity and larger error bars at 1 and 2 mm are due to the inclusion of points which are shifted in the gun‐target direction.

The relationship between MR/RT offset and MLC position error to detector dosimetric difference was characterized and correlated well with R^2^ = 0.99. One limitation of the technique is that MLC, MR/RT isocentricity, and dosimetric output are not decoupled. The dosimetric output could easily be decoupled by normalizing the 0.40 cm^2^ x 0.83 cm^2^ result by the daily dosimetric constancy acquired at 10.04 cm^2^ x 9.96 cm^2^. For the longitudinal assessment of MR/RT isocentricity and MLC accuracy, the impact of dosimetric uncertainty can be considered negligible as the output constancy for the 10.04 cm^2^ x 9.96 cm^2^ field was stable at 0.992 ± 0.012.

Our MLC positional accuracy is in agreeance to our institutional weekly QA MLC positional reproducibility.^9^ Small MLC gravitational‐induced effects combined with isocenter dependency with gantry rotation are observed, and are amplified due to the high sensitivity of the small‐field (i.e., 0.40 cm^2^ x 0.83 cm^2^) daily QA procedure. Our weekly MLC QA^9^ is performed for each MLC bank evaluated independently. A similar technique could be employed for the daily QA in which the four cardinal angles are measured twice for each upper and lower MLC bank, (i.e., MLC1 and MLC2) to allow for bank differences to be uniquely determined.

A limitation of the sensitivity of MR/lasers and IGRT positioning and repositioning evaluation is the spatial resolution of the imaging sequence. We used the highest available resolution at 1.5 mm^3^ x 1.5 mm^3^ x 1.5 mm^3^. Due to volume averaging across voxels and gantry dependency of the MRI, some resulting deviations were greater than 1 mm, however, 99.4% of measurements remained less than 2 mm. This approach of setting the gantry angle during MR imaging to a specified location to minimize MR/RT isocentricity has been implemented clinically at our institution for the initial 3D volumetric scan for MRgRT patient setup and simulation images.^15^ For IGRT position–reposition accuracy, a greater drift was noted at 10/2018 to 01/2019 (Fig. 8), likely attributed to interuser dependence of phantom localization, as timepoint corresponds to a rotation of operator. Although not performed for this study, mitigation of MRI gantry dependence can be performed through 3D volumetric imaging at the optimal gantry angle in which the centroid of MR isocenter is equal to the centroid of RT isocenter. Such an approach has been implemented clinically for the initial 3D volumetric scan for MRgRT setup and simulation images at our institution.

Additional unique considerations for MRgRT systems include fidelity of the MR imaging system, that is, spatial integrity and overall functionality of the MRI scanner. Verification of the absence of any ferromagnetic objects being lodged in the MRI scanner and communication between the MRI scanner and radiotherapy user interface are necessary components of the daily QA. Through our implemented technique, the daily QA serves as an end‐to‐end procedure in the clinical treatment workflow, therefore enabling evaluation that the TPDS and MRI scanner are communicating in the clinical state. An additional consideration with regard to the overall health of the MRI scanner is the integrity of the MRI receiver coils. At our institution a weekly procedure to verify the signal to noise (SNR) and percentage integral uniformity for coil integrity is performed. One could easily incorporate coil robustness by measuring the SNR over the uniform water areas of the phantom for the daily QA procedure.

A limitation of our technique is that the real‐time tracking and online adaptive components are not incorporated. Both mechanisms are likely low failure frequency as has been demonstrated over institutional practice and QA. Nonetheless, QA of these components should be established in a routine QA program. One practical method to verify treatment planning system integrity for online adaptive planning is incorporating a checksum to identify unintentional modifications to system configurations and database contents.^4^, ^16^ Detailed account of an end‐to‐end validation of online adaptive radiotherapy has been previously described by Mittauer *et al*. for an MRgRT program.^17^ Quality assurance of the gating capabilities of the MRIdian can be monitored with an end‐to‐end procedure with a motion phantom as described by Lamb *et al*.^18^

Although not part of TG142 criteria for daily QA, a limitation of our technique is that energy is not verified on a daily basis. One method to incorporate an energy check into the existing phantom design is to add a second ionization chamber at the IEC‐X landmark, that is, chamber holder. Here, the ratio of the distal chamber to the central chamber for the 10.04 cm^2^ x 9.96 cm^2^ field at G0 could be evaluated as a surrogate for energy constancy.

## 5 CONCLUSION

The linac and IGRT accuracy of a MR‐guided radiotherapy linac system been validated and monitored over seven months with an efficient, highly sensitive and robust method for routine QA. Longitudinal assessment demonstrated a drift in dose rate, but temporal assessment of output, MLC position, and isocentricity has been stable.

## CONFLICTS OF INTEREST

Dr. Mittauer reports personal fees and consulting fees from ViewRay Inc., ownership in MR Guidance, LLC. during the conduct of the study; Dr. Bayouth reports personal fees, consulting fees, and membership of Advisory Board of ViewRay Inc., ownership in MR Guidance, LLC during the conduct of the study.
